# The future of eye care in a changing world: call for papers

**DOI:** 10.2471/BLT.17.202952

**Published:** 2017-10-01

**Authors:** Alarcos Cieza, Ivo Kocur, Silvio Mariotti, Megan McCoy

**Affiliations:** a Management of Noncommunicable Diseases, Disability, Violence and Injury Prevention, World Health Organization, World Health Organization, avenue Appia 20, 1211 Geneva 27, Switzerland.

Eyesight plays a critical role in health and in people achieving a good quality of life. It has a pivotal influence on the way a person relates to and integrates into society, and an impact on many other areas, such as education and employment.^1^^–^^3^ Throughout the life course, vision affects child cognitive development, mental health, professional and personal trajectories and functional capacity in older people.^4^^–^^7^

Past investments in blindness prevention programmes have improved outcomes for individuals and generated economic benefits through enabling people to work – those directly affected and their caregivers.^8^^,^^9^ The age-standardized prevalence of severe, moderate and mild vision impairment is no longer increasing significantly, reflecting a shift in causes from communicable to chronic diseases.^10^

However it is estimated that population growth and ageing could contribute towards a tripling in the number of people with vision impairment; by 2050 there could be 115 million people who are blind, up from 38.5 million in 2020.^10^ New estimates also show that approximately 1 billion people over 35 years are currently affected by near vision impairment due to uncorrected presbyopia, 668 million of whom are over 50 years.^10^

However prevalence statistics only tell part of the story. Many people are affected by diseases or conditions that impact their vision and do not have timely access to services. There are also discrepancies in how different populations are affected. For example, women are estimated to have higher prevalence of blindness than men across all regions of the world.^10^In Australia, Aboriginal and Torres Strait Islander people have six times the rate of blindness of other Australians.^11^ There is however, a dearth of evidence about which groups miss out, why and what can be done about it.^12^

Eye care is often not well integrated into health systems, and often receives insufficient attention in workforce strategies and health information management systems, for example. Some health systems are therefore supporting service delivery models and approaches that may not be the most effective. Ineffective service delivery impacts on efficiency, reducing opportunities to free up and reallocate resources that could be used to improve quality or to reach groups that miss out. These resources are substantial; the annual global health system costs of recognizing, preventing and treating visual impairment have been estimated to be US$ 2.3 trillion.^13^

Increased efforts to provide timely and high-quality comprehensive eye care are needed in the context of population growth, non-communicable diseases and ageing. These demographic trends will lead to increased numbers of people with preventable and or irreversible vision loss. Eye care needs to be an integral part of universal health coverage to achieve the Sustainable Development Goals, in particular Goal 3 - ensure healthy lives and promote well-being for all at all ages.^14^

The *Bulletin of the World Health Organization* will publish a theme issue on vision. Papers for all sections of the Bulletin are welcomed around the central theme of ‘what works’. The theme issue will also supplement a forthcoming *World report on vision*. The report is expected to provide evidence on the prevalence and magnitude of eye diseases/conditions and vision loss globally, as well as its prevention, treatment and rehabilitation. It will offer recommendations, including a number focused on ensuring universal access to quality comprehensive and integrated eye care services in countries.

We welcome papers for the theme issue that provide evidence across all health strategies (promotion, prevention, treatment and rehabilitation) and systems building blocks, in particular, those that focus on best practices, innovation and the use of technology. Papers that identify gaps and provide solutions to ensure equitable access to services are encouraged. Papers should seek to integrate examples from low- and middle-income countries and different age groups. We strongly encourage papers that address health system issues, rather than focusing solely on one specific disease or condition. The deadline for submissions is 15 March 2018. Manuscripts should be submitted in accordance with the *Bulletin’s Guidelines for contributors* (http://submit.bwho.org), and the cover letter should mention this call for papers.
